# Hemodynamic Adaptation and Cardiac Effects of High-Flow Arteriovenous Access in Hemodialysis Patients: A Prospective Study

**DOI:** 10.3390/jcm14134556

**Published:** 2025-06-26

**Authors:** Yaeni Kim, Ji-hye Kim, Juyeon Woo, Oknan Choi, Mina Lee, Sunryoung Choi

**Affiliations:** 1 Department of Internal Medicine, Transplantation Research Center, College of Medicine, The Catholic University of Korea, Seoul 06591, Republic of Korea; yaeni@catholic.ac.kr; 2 Division of Nephrology, Department of Internal Medicine, Dialysis Access Center, Sahmyook Medical Center, 82 Mangu-ro, Dongdaemun-gu, Seoul 02500, Republic of Korea; 3 Department of Thoracic and Cardiovascular Surgery, Dialysis Access Center, Sahmyook Medical Center, 82 Mangu-ro, Dongdaemun-gu, Seoul 02500, Republic of Korea

**Keywords:** hemodialysis, arteriovenous access, cardiac output

## Abstract

**Background**: A vascular access blood flow (Qa) to cardiac output (CO) ratio greater than 0.3 increases the risk of high-output heart failure (HOHF). This study analyzed the characteristics and risk factors associated with a high Qa/CO ratio and effective CO (COef) in these patients over one year. **Methods**: This prospective study enrolled 142 hemodialysis (HD) patients, divided by the Qa/CO ratio. Baseline and one-year hemodynamics were measured: volume by bioimpedance, CO by echocardiography, Qa and brachial flow by transonic and ultrasound. Risk factors for HOHF were analyzed using receiver operating characteristic (ROC) curves and multivariable regression. **Results**: The study population was 66% male, mean age of 65, with 71% using arteriovenous fistulae (AVF) for vascular access. In the high Qa/CO group, cases of younger ages (62 vs. 67 years, *p* = 0.040) with upper arm access (73.1% vs. 32.8%, *p* < 0.001) were more prevalent, and diastolic blood pressure (DBP) was higher (76.8 ± 15.1 vs. 66.7 ± 14.4 mmHg, *p* = 0.002). Independent risk factors for high Qa/CO were elevated DBP (odds ratio [OR]: 1.080; 95% confidence interval [CI]: 1.028–1.134), upper arm access (OR: 8.113; 95% CI: 1.842–35.741), low resistive index (RI) (OR: 0.000; 95% CI: 0.000–0.417), and COef (OR: 0.164; 95% CI: 0.065–0.416). After one year, the high Qa/CO group showed significant increases in CO and COef (4.82 ± 1.25 L/min vs. 6.16 ± 2.05 L/min, *p* = 0.007, and 2.84 ± 0.95 L/min to 4.40 ± 1.89 L/min, *p =* 0.001, respectively). A baseline Qa cutoff value of 1.4 L/min demonstrated high diagnostic efficacy in identifying the high Qa/CO group. **Conclusions**: High Qa/CO ratios increase overt HOHF risk due to cardiac strain. This study reveals AV access-driven cardiac adaptations in patients with high Qa/CO and low COef, emphasizing the necessity for ongoing clinical and hemodynamic monitoring.

## 1. Introduction

The adoption and development of techniques to establish arteriovenous fistula (AVF) present a viable option for hemodialysis (HD) therapy. Vascular access blood flow (Qa) of 250 mL/min, as outlined in several guidelines, is required to achieve HD adequacy [1,2]. It is necessary to monitor and conduct surveillance of AVF with low Qa as this may suggest the presence of stenosis in the vascular conduit and forecast the development of thrombosis. In terms of vascular access patency as well as patient survival, the choice and maintenance of AVF over HD catheter and arteriovenous graft (AVG) demonstrated favorable outcomes [3].

However, in AVFs with a low-resistance venous pathway, it follows the consideration of balancing pressure and flow, not only to prevent thrombosis and ensure maturation, but also to avoid excessive flow that can lead to high-flow access complications. In a certain subset of patients where the maladaptive balance between vascular remodeling cannot cope with access flow and pressure, these immediate effects within one week of AVF creation can perpetuate further, entailing cardiac structural and functional damages, termed high-output heart failure (HOHF) [4].

HOHF is defined as the state of cardiac output (CO) greater than 8 L/min or cardiac index (CI) greater than 4 L/min, where heart failure results from its inability to keep up with abnormally high peripheral demand for blood flow [4,5]. The measured CO includes Qa in patients with AVFs and AVGs, leading to the concept of “effective cardiac output (COef),” calculated as the difference between CO and Qa [6]. The condition is associated with normal or near-normal cardiac function initially, but with decreased systemic vascular resistance (SVR), the increased workload can lead to cardiac remodeling and eventual systolic dysfunction [7,8]. HD patients with high Qa and CO are at increased risk for heart failure due to the increased volume and pressure load on the heart. A Qa/CO ratio greater than 0.30 (or 30%) has been suggested as a screening tool to identify patients at risk of developing HOHF as this ratio represents the proportion of CO that is being shunted through the AVF [9]. However, the characteristics and risk factors associated with the development of high Qa/CO in HD patients are not well understood. Additionally, the hemodynamic processes involved in the high-output shunting of blood in the pathogenesis of HOHF remain unclear. It is important to note that while this ratio is used as a screening tool, it may not be the most accurate predictor of HOHF. Some other parameters, such as estimated SVR, appear to be more predictive of HOHF hospitalization than the Qa/CO ratio [10].

In this study, we aimed to identify factors associated with the development of HOHF with high Qa/CO by examining various cardiac and vascular access parameters using ultrasound in prevalent HD patients and observing these changes over one year.

## 2. Materials and Methods

### 2.1. Study Population and Design

This study was conducted at Sahmyook Medical Center (Seoul, Republic of Korea). One hundred forty-two maintenance HD patients were followed up from March 2021 to February 2023. HD was performed three times weekly for four hours each time. Patients with a history of cardiovascular disease and transplantation, with active infection, with malignancy, and those who failed to undergo an echocardiogram were excluded. Specifically, we excluded patients with documented coronary artery disease, including prior myocardial infarction, percutaneous coronary intervention, or coronary artery bypass grafting, a clinical or echocardiographic diagnosis of heart failure, significant valvular heart disease, or known cardiomyopathy. To define echocardiographic evidence of heart failure, we excluded patients with any of the following: moderate to severe left ventricular systolic dysfunction, defined as LV ejection fraction (LVEF < 40%), Grade 3 or higher left ventricular diastolic dysfunction, based on guideline-recommended parameters, presence of arrthyrhmias, such as atrial fibrillation, at the time of echocardiography, moderate to severe valvular regurgitation or stenosis, significant wall motion abnormalities consistent with ischemic heart disease. Patients were grouped based on the level of Qa/CO ratio: a ratio exceeding 0.3 was classified as high, while a ratio less than 0.3 was classified as normal. This prospective observational study was conducted in accordance with the principles contained in the Declaration of Helsinki and was approved by the Institutional Review Board of Sahmyook Medical Center (IRB no. 116286-202206-HR-03). Although the study was conducted prospectively in terms of data collection during the observational period (March 2021–February 2023), it was analyzed retrospectively. The data were derived from previously recorded clinical information and routine documentation collected as part of standard patient care, without any experimental intervention or deviation from clinical practice. As such, the study posed minimal risk to participants and did not alter standard clinical care. The IRB approval pertains to the retrospective analysis protocol. This approval was granted after the observational data collection had commenced, as IRB review was only necessary at the point of retrospective evaluation. A waiver of informed consent was also granted due to the retrospective nature of the study and the absence of any patient contact or additional data collection efforts beyond routine clinical practice.

### 2.2. Clinical and Biochemical Parameters

Baseline parameters, including demographic, laboratory, and HD data, were collected at the start of the study. Blood samples were obtained before the midweek dialysis session to measure various markers using standard techniques [11]. Body mass index (BMI) was calculated as dry weight in kilograms divided by height in meters squared (kg/m^2^). Kt/V was calculated using the logarithmic estimate of the Daugirdas method [12]. Dry weight was ascertained by clinical assessment and using bioimpedance and chest X-ray at least monthly [11].

### 2.3. Bioimpedance Methodology

The Body Composition Monitor (BCM) expresses body composition as a three-compartment model, providing overhydration (OH), lean tissue index (lean tissue mass/height^2^, LTI), and fat tissue index (adipose tissue mass/height^2^, FTI), respectively [13]. The three-compartment model of the BCM has been validated for fluid status and body composition in HD patients [14,15]. BCM measurements were performed before HD.

### 2.4. Echocardiography

Transthoracic echocardiograms were performed for baseline assessments on predialysis and obtained by fundamental imaging (two-dimensional, M-mode, and tissue Doppler imaging (TDI)) using a 2.5 MHz transducer and commercial ultrasound system (Vivid 9, GE Vingmed Ultrasound AS, Horten, Norway). Chamber dimension, wall thickness, and left ventricular (LV) ejection fraction (EF) were measured (M-mode), and the mitral annular velocities were obtained by TDI. Left atrial dimension (LAD) was determined from M-mode echocardiograms using a leading edge to leading edge technique [16]. We measured the peak velocities of early diastole (e′) and calculated the E/e′ ratio. The E/e′ ratio reflects the mean LV diastolic pressure, and the E/e′ ratio > 15 indicates LV diastolic dysfunction (LVDD) [17]. The LV mass was calculated using the Devereux formula [LV mass (g) = 1.04 × (interventricular septal thickness + LV end-diastolic dimension + posterior wall thickness)^3^ − (LV end-diastolic dimension)^3^ − 13.6] [18], and the LV mass index (LVMI) was calculated as LV mass in grams divided by body surface area [19]. The LV systolic function was assessed by calculating the EF using a modified Simpson’s method [20]. The left ventricular outflow tract (LVOT) diameter was measured in the parasternal long-axis view during mid-systole, approximately 0.5 cm from the aortic valve leaflet insertion points. LVOT velocity time integral (VTI) was obtained using pulsed-wave Doppler in the apical five-chamber view, with the sample volume positioned just proximal to the aortic valve. The LVOT cross-sectional area was calculated using the formula π (LVOT diameter/2)^2^. Stroke volume was determined by multiplying the LVOT cross-sectional area by the LVOT VTI. Cardiac output (CO) was then calculated as the product of stroke volume and heart rate [21]. All measurements were performed in triplicate and averaged to ensure accuracy. Echocardiography was performed by one experienced specialist who was blinded to the information about the patients. We also calculated the Cardiac Index (CI) as it is considered a more accurate measure of cardiac performance than CO, as it takes into account the variation in body size between patients (CI = CO/BSA).

### 2.5. Access Flow (Qa) Measurement

Qa was measured three times with the ultrasound dilution HD03 HD monitor (Transonic Systems, Ithaca, NY, USA) based on an ultrasound dilution technique previously described [22].

### 2.6. Duplex Ultrasound Measurement

The time-averaged velocity integral of the mean velocity (TAVM) and the brachial artery flow rate (BAFR) were measured in the brachial artery, at least 5 cm proximal to the anastomosis or above the elbow, by the recommended method [23]. Peak systolic velocity (PSV), end diastolic velocity (EDV), TAVM, pulsatility index (PI), resistive index (RI), acceleration, and acceleration time (AT) were calculated using the Doppler spectrum for AV access flow measurement in the brachial artery.

### 2.7. Statistical Analysis

Summary statistics are expressed as the mean (standard deviation) or median (interquartile range) for continuous variables and as frequencies or percentages for categorical variables. Continuous variables were compared using Student’s *t*-test for two groups or the Mann–Whitney U test for non-parametric tests. Categorical variables were compared using the Chi-square test. Multivariate analysis by the enter method was performed on variables that were shown to be meaningful in the univariate analysis. Receiver operating characteristic (ROC) curves were performed to identify predictable Qa and brachial artery flow cutoffs and Qa/CO ratio. All tests were performed using SPSS, version 18.0 (SPSS, Inc., Chicago, IL, USA). A *p*-value of less than 0.05 was considered significant.

## 3. Results

### 3.1. Baseline Characteristics According to Qa/CO Ratio

Table 1 shows baseline characteristics of patients according to the Qa/CO ratio. Among the total 142 participants, the number of patients with a high Qa/CO ratio (Qa/CO > 0.3) was 26 (18.3%). The mean age was 65.9 ± 12.0 years, and 94 patients (66.1%) were men. The median dialysis vintage was 65.9 months. A total of 104 patients were receiving HD using AVF (73.2%), 57 patients had upper arm AV access (40.1%), and the patients in the group with increased Qa/CO had significantly higher upper arm access (73.1% vs. 32.8%, *p* < 0.001). The high Qa/CO group had significantly higher levels of pre-dialysis diastolic blood pressure (DBP) (76.8 ± 15.1 mmHg vs. 66.7 ± 14.4 mmHg, *p* = 0.002) and higher access flow rate (Qa) (1979.6 ± 510.5 mL/min vs. 930.4 ± 342.9 mL/min, *p* < 0.001) than the normal Qa/CO group. No significant differences were observed in the BMI (24.4 ± 3.8 kg/m^2^ vs. 22.9 ± 5.2 kg/m^2^, *p* = 0.087) and Kt/V (1.64 ± 0.26 vs. 1.70 ± 0.23, *p* = 0.313) between the normal Qa/CO and high Qa/CO ratio groups, respectively.

### 3.2. Bioimpedance Parameters According to Qa/CO Ratio

Table 2 shows a comparative analysis of BCM in the normal Qa/CO and high Qa/CO ratio groups. No significant difference was observed in overhydration (OH) (2.54 ± 2.10 L vs. 2.90 ± 2.20 L, *p* = 0.433), extracellular water/total body water (ECW/TBW) ratio (15.0 ± 10.4% vs. 17.2 ± 9.5%, *p* = 0.337), lean tissue index (LTI) (13.9 ± 3.5 kg/m^2^ vs. 12.8 ± 2.7 kg/m^2^, *p* = 0.141), and fat tissue index (FIT) (9.4 ± 4.5 kg/m^2^ vs. 9.4 ± 6.8 kg/m^2^, *p* = 0.999) between the two groups.

### 3.3. Baseline Echocardiographic Findings According to Qa/CO Ratio

Table 3 demonstrates baseline echocardiographic findings according to the Qa/CO ratio. The high Qa/CO group had significantly lower levels of CO (4.82 ± 1.25 vs. 5.77 ± 1.58 L/min, *p* = 0.005), CI (2.96 ± 0.60 vs. 3.46 ± 0.88 L/min/m^2^, *p* = 0.007), and COef (2.84 ± 0.95 vs. 4.86 ± 1.54 L/min, *p* < 0.001) than the normal Qa/CO group. There was no difference in the E/e′ ratio, LVMI, and EF between the two groups, and there was no difference in the proportion of patients with LVDD, LVH, and LVSD. LAVI, RSVP, and CO + Qa were also not different between the two groups.

### 3.4. Access Flow and Duplex Ultrasound Parameters of Vascular Access According to Qa/CO Ratio

**Figure 1:**
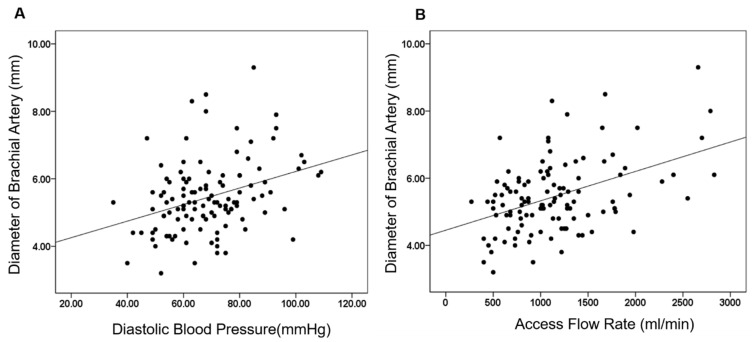
Correlations between brachial artery diameter, diastolic blood pressure, and vascular access flow. The scatter plots demonstrate statistically significant positive correlations between DBP and brachial artery diameter (**A**), and between vascular access flow (Qa) and brachial artery diameter (**B**) (*r* = 0.342, *p* < 0.001, *r* = 0.443, *p* < 0.001, respectively).

Table 4 demonstrates access flow measured by Transonic and baseline ultrasonographic findings of various HD vascular access parameters according to the Qa/CO ratio. The group with high Qa/CO had significantly higher Qa (1979.6 ± 510.5 vs. 930.4 ± 342.9 mL/min, *p* < 0.001) than the group with normal Qa/CO. Brachial artery flow was also significantly higher in the group with elevated Qa/CO (1677.4 ± 401.8 vs. 938.8 ± 327.0 mL/min, *p* < 0.001). Patients with increased Qa/CO had significantly higher values for PSV (239.6 ± 69.1 vs. 199.5 ± 69.2 cm/s, *p* = 0.009), EDV (139.8 ± 48.1 vs. 99.9 ± 42.2 cm/s, *p* < 0.001), TAMV (101.6 ± 40.3 vs. 76.9 ± 33.0 cm/s, *p* = 0.001), and brachial artery diameter (6.02 ± 1.29 vs. 5.26 ± 0.98 mm, *p* = 0.001), while RI was significantly lower (0.43 ± 0.10 vs. 0.50 ± 0.09, *p* < 0.001). Figure 1 demonstrates correlations between brachial artery diameter, DBP, and vascular access flow. The scatter plots demonstrate statistically significant positive correlations between DBP and brachial artery diameter, and between Qa and brachial artery diameter (*r* = 0.342, *p* < 0.001, *r* = 0.443, *p* < 0.001, respectively).

### 3.5. Univariate and Multivariate Logistic Regression Analyses for High Qa/CO Ratio

Table 5 illustrates univariate and multivariate analysis results for investigating factors associated with high Qa/CO. Multivariate logistic regression analysis revealed that upper arm access (odds ratio [OR]: 8.113; 95% confidence interval [CI]: 1.842–35.741), high pre-dialysis DBP (OR: 1.080; 95% CI: 1.028–1.134), low RI (OR: 0.000; 95% CI: 0.000–0.417), and COef (OR: 0.164; 95% CI: 0.065–0.416) values were independent risk factors for high Qa/CO ratio.

### 3.6. Receiver Operating Characteristic (ROC) Curve for High Qa/CO Ratio

**Figure 2:**
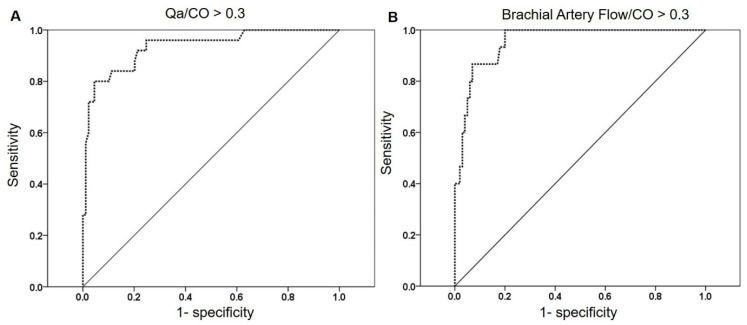
Receiver operating characteristic (ROC) curve analyses to determine optimal cutoff values for predicting a high Qa/CO ratio (>0.3). Access flow (Qa) >1385 mL/min (AUC = 0.968, sensitivity 90.5%, specificity 90.3%, *p* < 0.001) (**A**) and brachial artery flow > 1404.5 mL/min (AUC = 0.955, sensitivity 86.7%, specificity 87%, *p* < 0.001) (**B**) showed excellent discriminatory power for identifying patients with a Qa/CO ratio exceeding 0.3.

ROC analyses were performed to estimate the best cutoffs to predict a high Qa/CO ratio. Our analysis showed that Qa (>1385 mL/min, AUC = 0.968, *p* < 0.001) and brachial artery flow (>1404.5 mL/min, AUC = 0.955, *p* < 0.001) offered reasonable sensitivity (90.5 vs. 86.7%) and specificity (90.3 vs. 87%) for Qa/CO over 0.3 (Figure 2).

### 3.7. Changes in Echocardiographic Parameters According to Qa/CO Ratio

Table 6 demonstrates changes in echocardiographic parameters after one year according to Qa/CO groups. In the high Qa/CO group, there were significant increases in CO (4.82 ± 1.25 vs. 6.16 ± 2.05 L/min, *p* = 0.007), CI (2.96 ± 0.60 vs. 3.71 ± 0.97 L/min/m^2^, *p* = 0.005), and COef (2.84 ± 0.95 vs. 4.40 ± 1.89 L/min, *p* = 0.001) parameters after one year compared to the baseline values. However, Qa significantly decreased after one year compared to the baseline value (1979.6 ± 510.5 vs. 1696.1 ± 645.3 mL/min, *p* < 0.001). In the low Qa/CO group, there were no significant differences in echocardiographic parameters between the baseline and one-year follow-up, except for a decrease in LAVI after one year (45.9 ± 15.7 vs. 42.9 ± 14.1 mL/m^2^, *p* = 0.035) (Supplementary Tables S1 and S2).

## 4. Discussion

Our study provides valuable insights into the vascular, cardiac, and volume status changes in HD patients with different Qa/CO ratios. Patients with a high Qa/CO ratio (>30%) were typically younger, non-diabetic, had higher DBP, and utilized upper arm access (brachiocephalic fistula) with increased Qa. At baseline, these patients exhibited lower CO, COef, and CI compared to those with a normal Qa/CO ratio. Over one year, CO, COef, and CI increased, while Qa decreased in the high Qa/CO group; no significant hemodynamic changes were observed in the low Qa/CO group. Factors associated with a high Qa/CO ratio included upper arm access, higher DBP, and lower RI and COef.

The hemodynamic effects of AVF creation on the heart are direct and prominent. Immediately post-creation, CO increases by 10 to 20% due to heightened sympathetic tone and decreased peripheral resistance. Within a week, increased blood volume leads to elevated left ventricular end-diastolic volume (LVEDV), neurohormones such as atrial natriuretic peptide (ANP) and brain natriuretic peptide (BNP), and decreased systemic vascular resistance (SVR), renin, and aldosterone. This further contributes to increased stroke volume and heart rate, accelerating cardiac remodeling [4,24,25]. However, not all cases progress to symptomatic HOHF. By the time symptoms appear and treatment is initiated, cardiac remodeling may have already become irreversible. Therefore, it is crucial to identify patients at high risk for HOHF before symptoms manifest. In this context, establishing the characteristics of a high Qa/CO ratio, which is associated with increased HOHF risk, becomes clinically significant.

In the high Qa/CO group, AVF flow (Qa) and the brachial artery blood flow rate were significantly higher, indicating greater shunting of blood through the fistula. This suggests a large, high-flow AVF that demands substantial arterial inflow, potentially impacting systemic circulation. Increased TAMV and brachial artery dilation reflect arterial remodeling in response to chronic high-flow conditions. A lower RI in the brachial artery suggests reduced downstream resistance, as the AVF acts as a low-resistance conduit, diverting a significant portion of blood flow, which may compromise systemic perfusion [26]. These findings are consistent with high-flow access characteristics and suggest that these patients are at a higher risk of developing high-flow-related heart failure.

Initially, excessive AVF flow diverts blood away from systemic circulation, reducing COef. Over time, the heart compensates by increasing total CO, likely through enhanced stroke volume and myocardial adaptation, leading to a gradual decline in the Qa/CO ratio as more CO is redirected toward systemic circulation [27]. However, despite expectations that a higher Qa should trigger a proportional rise in CO to maintain systemic perfusion, the high Qa/CO group paradoxically exhibited lower CO, CI, and COef, while Qa + CO remained similar between groups. This paradox may be explained by multiple factors: the steal phenomenon, increased afterload, and impaired autonomic regulation. The fact that Qa + CO remained unchanged between groups suggests that while the total hemodynamic burden on the heart was similar, the distribution of blood flow differed, with the high Qa/CO group experiencing compromised systemic perfusion.

Echocardiographic changes over one year provide insights into cardiovascular adaptation and remodeling in response to AVF-related hemodynamic shifts. In the normal Qa/CO group, LAVI significantly decreased, suggesting improved left atrial compliance and reduced volume overload. At baseline, patients with a high Qa/CO ratio demonstrated a low CIef (1.75 L/min/m^2^), suggesting systemic hypoperfusion. However, over time, CIef improved to 2.66 L/min/m^2^, approaching normal levels. This improvement could be explained by gradual hemodynamic adaptation, where the cardiovascular system compensates for the high-flow AVF by either reducing Qa over time or increasing SVR or decreasing access resistance. The AVF may undergo autoregulatory structural remodeling, leading to a relative decrease in flow, thereby improving CIef. This led to subsequent increases in CO, CI, and COef. Since Qa and Qa/CO decreased, the heart may have redistributed more blood toward systemic circulation, resulting in higher effective perfusion. SVR may increase as a compensatory response to excessive AVF flow. The increase in DBP supports this hypothesis, as it suggests a rise in afterload due to systemic vasoconstriction. Another possible explanation for Qa reduction over time is the development of AV access outflow stenosis, which can gradually increase access resistance. This would restrict access flow, leading to a relative increase in systemic perfusion (higher CO, CI, and COef). Despite these hemodynamic alterations, bioimpedance analysis revealed no significant differences in water balance between groups. This suggests that increased access flow did not translate into overt volume overload, potentially due to effective ultrafiltration strategies during dialysis or compensatory venous return mechanisms.

Subgroup analysis further revealed distinct hemodynamic differences between patients with AVFs and those with grafts. In the AVF group, Qa remained stable over time, while CO, CI, and COef increased (Supplementary Table S3). Conversely, patients with grafts exhibited a different pattern, in which a reduction in Qa was not associated with significant changes in CO and CI (Supplementary Table S4). This may reflect the ability of native AVFs to maintain their patency and autoregulate blood flow more effectively than grafts, owing to their superior vascular adaptation to chronic high-flow conditions. Unlike native AVFs, synthetic grafts lack the capacity for vascular remodeling and autoregulation, limiting their ability to adapt to changes in blood flow, which may dampen the effects of Qa changes on total CO [28]. These distinctions underscore the importance of individualized monitoring and management strategies based on the type of vascular access.

The increases in DBP, along with decreases in COef and RI in those with upper arm access, were factors associated with a high Qa/CO ratio. Our regression analysis reveals that increases in DBP and Qa are associated with a concomitant increase in brachial artery diameter, suggesting a potential relationship between peripheral vascular resistance and arterial remodeling in this patient cohort (Figure 1). The diameter of the Inflow artery, along with heart rate and BP, determines the potential AVF flow volume. After AVF creation, blood flow rates in the brachial artery can increase 10- to 80-fold, rising from a baseline of approximately 50 mL/min due to a significant reduction in vascular resistance [29]. Over time, changes in shear stress promote outward arterial remodeling, further increasing flow volume in the dialysis access circuit. Conversely, arteries with diameters smaller than 2 mm may restrict blood flow to less than 400 mL/min, depending on BP [30]. This suggests that selecting arteries with smaller diameters for AVF creation and implementing strict BP control may help mitigate the risk of HOHF by limiting AVF flow volume. To predict this risk, a brachial artery flow rate of 1400 mL/min or higher has been identified as a useful diagnostic indicator for Qa/CO values exceeding 0.3. This threshold may help clinicians identify patients at risk for HOHF earlier and implement targeted preventive measures.

While our study has limitations, including a relatively small sample size, homogeneity of the study cohorts, and being a single-center study with a one-year observation period, it offers significant insights as a prospective cohort study. Clinically, many patients with high Qa remain asymptomatic, raising questions about the necessity and timing of intervention. Our findings offer a possible explanation: in high-flow patients, while Qa decreases over time, COef tends to increase. This compensatory increase in COef may help preserve overall cardiac function, potentially explaining why these patients often remain asymptomatic and show no significant changes in other echocardiographic parameters. However, patients who do not exhibit an increase in COef despite decreasing Qa may warrant closer monitoring, as they could be at higher risk for subclinical or progressive cardiac dysfunction. A longer follow-up period might have allowed us to determine whether these “non-compensators” ultimately experience cardiac deterioration.

In conclusion, the complex interplay of high Qa/CO ratios and low COef suggests that persistently low COef in high-flow patients may serve as an early indicator of impaired cardiac adaptation. Moreover, surrogate markers of SVR, including DBP and RI, when considered alongside their inverse relationship with COef, further support this evolving paradigm and may aid risk stratification. These patients may benefit from flow-reduction interventions even in the absence of symptoms. Future prospective studies with extended follow-up are needed to validate this hypothesis and to help define optimal timing for intervention

## Figures and Tables

**Table 1:** Baseline characteristics of patients according to Qa/CO ratio.

	All (n = 142)	Qa/CO ≤ 0.3 (n = 116)	Qa/CO > 0.3 (n = 26)	*p* Value
Demographic data				
Age, years	65.9 ± 12.0	67.4 ± 11.5	62.0 ± 14.1	0.040
Male, n (%)	94 (66.1)	79 (68.1)	15 (57.7)	0.361
DM, n (%)	102 (71.8)	88 (75.9)	14 (53.8)	0.031
Hypertension, n (%)	132 (93.0)	110 (94.8)	22 (84.6)	0.085
Pre-dialysis SBP, mmHg	154.2 ± 23.6	152.8 ± 22.9	158.9 ± 27.3	0.239
Pre-dialysis DBP, mmHg	69.2 ± 15.1	66.7 ± 14.4	76.8 ± 15.1	0.002
Pulse rate, rate/min	72.4 ± 11.7	71.7 ± 11.3	73.6 ± 12.5	0.461
Dialysis vintage, months	65.9 ± 47.1	66.9 ± 47.8	64.9 ± 48.6	0.845
BMI, kg/m^2^	24.2 ± 4.1	24.4 ± 3.8	22.9 ± 5.2	0.087
Kt/V	1.65 ± 0.25	1.64 ± 0.26	1.70 ± 0.23	0.313
Fistula, n (%)	104 (73.2)	80 (69.6)	21 (80.8)	0.337
Upper arm access	57 (40.1)	38 (32.8)	19 (73.1)	<0.001
Access flow, Qa (mL/min)	1132.5 ± 571.6	930.4 ± 342.9	1979.6 ± 510.5	<0.001

DM, diabetes mellitus; BMI, body mass index; SBP, systolic blood pressure; DBP, diastolic blood pressure.

**Table 2:** Bioimpedance parameters according to Qa/CO ratio.

	All (n = 142)	Qa/CO ≤ 0.3 (n = 116)	Qa/CO > 0.3 (n = 26)	*p* Value
Overhydration, L	2.63 ± 2.13	2.54 ± 2.10	2.90 ± 2.20	0.433
TBW, L	32.6 ± 7.2	33.0 ± 7.3	30.5 ± 7.5	0.129
ECW/TBW ratio, %	15.3 ± 10.4	15.0 ± 10.4	17.2 ± 9.5	0.337
LTI, kg/m^2^	13.7 ± 3.3	13.9 ± 3.5	12.8 ± 2.7	0.141
FTI, kg/m^2^	9.4 ± 5.0	9.4 ± 4.5	9.4 ± 6.8	0.999

TBW: total body water, ECW: extracellular water, LTI: lean tissue index, FTI: fat tissue index.

**Table 3:** Baseline echocardiographic findings according to Qa/CO ratio.

	All (n = 142)	Qa/CO ≤ 0.3 (n = 116)	Qa/CO > 0.3 (n = 26)	*p* Value
LAD, cm	4.10 ± 0.58	4.07 ± 0.57	4.02 ± 0.65	0.706
LAVI, mL/m^2^	46.5 ± 16.1	46.1 ± 15.3	50.1 ± 20.6	0.263
RVIDd, mm	3.02 ± 0.40	3.04 ± 0.41	3.03 ± 0.32	0.913
IVSd, mm	1.08 ± 0.20	1.10 ± 0.18	1.00 ± 0.25	0.064
IVSs, mm	1.40 ± 0.23	1.41 ± 0.22	1.36 ± 0.22	0.281
LVIDd, mm	4.78 ± 0.66	4.70 ± 0.67	4.97 ± 0.61	0.076
LVIDs, mm	3.31 ± 0.63	3.23 ± 0.63	3.48 ± 0.56	0.064
LVPWd, mm	1.07 ± 0.16	1.07 ± 0.15	1.06 ± 0.19	0.686
LVPWs, mm	1.40 ± 0.21	1.39 ± 0.19	1.38 ± 0.19	0.911
E, cm/s	92.9 ± 27.0	92.2 ± 28.0	97.1 ± 24.0	0.415
A	98.1 ± 25.6	99.5 ± 27.3	92.8 ± 18.2	0.143
E/A	1.01 ± 0.52	1.01 ± 0.57	1.05 ± 0.27	0.753
RWT, mm	0.45 ± 0.10	0.46 ± 0.10	0.43 ± 0.09	0.128
RVSP, mmHg	31.0 ± 14.3	30.3 ± 14.0	34.6 ± 14.8	0.165
LVM, g	191.0 ± 54.1	187.5 ± 51.5	196.4 ± 59.4	0.440
LVMI, g/m^2^	114.2 ± 29.3	112.1 ± 28.7	120.1 ± 30.8	0.202
LVH, n (%)	32 (23.9)	23 (21.1)	9 (36.0)	0.125
E/e′ ratio	14.5 ± 5.1	14.5 ± 5.1	14.9 ± 5.0	0.770
LVDD, n (%)	63 (44.7)	51 (44.0)	12 (48.0)	0.825
EF, %	57.7 ± 8.0	57.8 ± 7.9	58.4 ± 6.9	0.722
LVSD, n (%)	19 (13.7)	16 (14.0)	3 (12.0)	1.000
CO, L/min	5.69 ± 1.56	5.77 ± 1.58	4.82 ± 1.25	0.005
CI, L/min/m^2^	3.36 ± 0.86	3.46 ± 0.88	2.96 ± 0.60	0.007
Effective CO, L/min	4.49 ± 1.65	4.86 ± 1.54	2.84 ± 0.95	<0.001
CO + Qa, L/min	6.73 ± 1.70	6.72 ± 1.72	6.80 ± 1.66	0.825
Qa/CO	0.21 ± 0.12	0.17 ± 0.06	0.42 ± 0.09	<0.001

RVID = RV internal dimension; LAD = left atrial dimension; LVID = left ventricular Internal dimension; IVS = Interventricular septum thickness; PW = posterior wall thickness; RWT = relative wall thickness; RVSP = RV systolic pressure; LVMI = Left ventricular mass index; LVH = Left ventricular hypertrophy; E = early diastolic mitral inflow velocity; e′ = early diastolic mitral annular velocity; LVDD = left ventricular diastolic dysfunction; EF = ejection fraction; LVSD = left ventricular systolic dysfunction; CO = cardiac output; CI = cardiac index. Qa = access flow; d = end diastolic; s = end systolic.

**Table 4:** Access flow and duplex ultrasound parameters of vascular access according to Qa/CO ratio.

	All (n = 142)	Qa/CO ≤ 0.3 (n = 116)	Qa/CO > 0.3 (n = 26)	*p* Value
Access flow, mL/min	1132.5 ± 571.6	930.4 ± 342.9	1979.6 ± 510.5	<0.001
Brachial artery				
PSV, cm/s	205.6 ± 70.2	199.5 ± 69.2	239.6 ± 69.1	0.009
EDV, cm/s	106.0 ± 45.1	99.9 ± 42.2	139.8 ± 48.1	<0.001
PI	0.75 ± 0.26	0.78 ± 0.25	0.59 ± 0.20	<0.001
RI	0.49 ± 0.10	0.50 ± 0.09	0.43 ± 0.10	<0.001
Acceleration, cm/s^2^	743.2 ± 359.4	743.8 ± 365.4	673.7 ± 253.0	0.363
AT, s	0.15 ± 0.05	0.15 ± 0.05	0.16 ± 0.04	0.254
TAMV, cm/s	80.7 ± 35.1	76.9 ± 33.0	101.6 ± 40.3	0.001
Diameter, mm	5.40 ± 1.09	5.26 ± 0.98	6.02 ± 1.29	0.001
Flow, mL/min	1067.2 ± 445.3	938.8 ± 327.0	1677.4 ± 401.8	<0.001

PSV: peak systolic velocity; EDV: end diastolic velocity; PI: pulsatility index; RI: resistive index; TAVM: time-averaged velocity integral of the mean velocity; AT: acceleration time.

**Table 5:** Univariate and multivariate logistic regression analyses for high Qa/CO ratio.

		Univariate			Multivariate	
	Beta	Odds Ratio (95% CI)	*p* Value	Beta	Odds Ratio (95% CI)	*p* Value
Age, years	−0.037	0.964(0.931~0.999)	0.043			
Hypertension	−1.204	0.300(0.078~1.152)	0.079			
Diabetes mellitus	−0.991	0.371(0.154~0.895)	0.027	0.562	1.754(0.376~8.120)	0.472
BMI, kg/m^2^	−0.111	0.895(0.788~1.016)	0.087			
Male gender	−0.488	0.639(0.267~1.525)	0.313			
Upper arm access	1.718	5.571(2.156~14.397)	<0.001	2.093	8.113(1.842~35.741)	0.006
Fistula	0.608	1.837(0.641~5.267)	0.257			
SBP, mmHg	0.011	1.011(0.993~1.030)	0.239			
DBP, mmHg	0.046	1.047(1.016~1.079)	0.003	0.077	1.080(1.028~1.134)	0.002
LVMI, g/m^2^	0.010	1.010(0.995~1.025)	0.263			
LAVI, mL/m^2^	0.014	1.014(0.989~1.040)	0.263			
Brachial artery						
RI	−10.706	0.000(0.000~0.007)	<0.000	−9.764	0.000(0.000~0.417)	0.031
Overhydration, L	0.048	1.049(0.862~1.277)	0.630			
Effective CO, L/min	−1.749	0.174(0.085~0.358)	<0.000	−1.808	0.164(0.065~0.416)	<0.001

BMI: body mass index; SBP: systolic blood pressure; DBP: diastolic blood pressure; LVMI: Left ventricular mass index; LAVI: left atrial volume index; RI: resistive index; CO: cardiac output.

**Table 6:** Changes in echocardiographic parameters according to Qa/CO ratio.

	Qa/CO ≤ 0.3		*p* Value	Qa/CO > 0.3		*p* Value
	Baseline	1 Year Later		Baseline	1 Year Later	
CO, L/min	5.81 ± 1.53	6.05 ± 2.32	0.287	4.82 ± 1.25	6.16 ± 2.05	0.007
CI, L/min/m^2^	3.50 ± 0.87	3.69 ± 1.46	0.184	2.96 ± 0.60	3.71 ± 0.97	0.005
Effective CO, L/min	4.90 ± 1.51	5.01 ± 2.22	0.299	2.84 ± 0.95	4.40 ± 1.89	0.001
Effective CI, L/min/m^2^	2.91 ± 0.85	3.10 ± 1.39	0.216	1.75 ± 0.50	2.66 ± 0.96	0.001
CO + Qa, L/min	6.77 ± 1.62	7.02 ± 2.51	0.288	6.80 ± 1.66	7.91 ± 2.36	0.053
Qa/CO	0.17 ± 0.06	0.17 ± 0.08	0.798	0.42 ± 0.09	0.30 ± 0.12	<0.001
Qa, mL/min	930.4 ± 342.9	949.9 ± 435.4	0.710	1979.6 ± 510.5	1696.1 ± 645.3	<0.001

## Data Availability

The raw data supporting the conclusions of this article will be made available by the authors on request.

## Supplementary Materials

The following supporting information can be downloaded at: https://www.mdpi.com/article/10.3390/jcm14134556/s1, Table S1. Changes in Echocardiographic Parameters in the High Qa/CO Group. Table S2. Changes in Echocardiographic Parameters in the Low Qa/CO Group. Table S3. Changes in Echocardiographic Parameters According to the Qa/CO Ratio Among Patients with Arteriovenous Fistulas. Table S4. Changes in Echocardiographic Parameters According to the Qa/CO Ratio Among Patients with Arteriovenous Graft.
