# Supplementary material for: Hemodynamic Adaptation and Cardiac Effects of High-Flow Arteriovenous Access in Hemodialysis Patients: A Prospective Study
Source: J Clin Med. 2025 Jun 26;14(13):4556. doi: 10.3390/jcm14134556 (PMC12249853; doi:10.3390/jcm14134556)
Supplement: Supplementary file 1 [file jcm-14-04556-s001.zip › jcm-3684078-supplementary.pdf]

Supplementary Table S1. Changes in Echocardiographic Parameters in the High Qa/CO Group

Parameters	Baseline	1 year later	*p* value
Aortic root	3.09±0.32	3.17±0.40	0.181
LAD, cm	4.02±0.65	4.10±0.56	0.785
LAVI, ml/m^2	50.1±20.6	52.1±21.6	0.969
RVIDd, mm	3.03±0.32	3.05±0.35	0.578
IVSd, mm	1.00±0.25	1.06±0.19	0.478
IVISs, mm	1.36±0.22	1.36±0.18	0.639
LVIDd, mm	4.97±0.61	4.92±0.69	0.384
LVIDs, mm	3.48±0.56	3.40±0.60	0.516
LVPWd, mm	1.06±0.19	1.04±0.16	0.364
LVPWs, mm	1.38±0.19	1.34±0.16	0.179
E, cm/s	97.1±24.0	89.0±28.4	0.121
A	92.8±18.2	90.7±19.5	0.155
E/A	1.05±0.27	0.99±0.42	0.674
RWT, mm	0.43±0.09	0.40±0.12	0.318
RVSP, mmHg	34.6±14.8	30.3±8.8	0.256
LVM, g	196.4±59.4	192.7±55.5	0.214
LVMI, g/m^2	120.1±30.8	116.6±29.3	0.258
E/e\` ratio	14.9±5.0	13.0±4.3	0.138
EF, %	58.4±6.9	59.0±5.1	0.596
CO, L/min	4.82±1.25	6.16±2.05	0.007
CI, L/min/m^2	2.96±0.60	3.71±0.97	0.005
Effective CO, L/min	2.84±0.95	4.40±1.89	0.001
Effective CI, L/min/m^2	1.75±0.50	2.66±0.96	0.001
Qa+CO, L/min	6.80±1.66	7.91±2.36	0.053
Qa/CO,	0.42±0.09	0.30±0.12	<0.001
Qa, ml/min	1979.6±510.5	1696.1±645.3	<0.001

RVID, RV internal dimension; LAD = left atrial dimension; LAVI=left atrial volume index; LVID, left ventricular Internal dimension; IVS, Interventricular septum thickness; PW = posterior wall thickness; RWT, relative wall thickness; RVSP, RV systolic pressure; LVMI = Left ventricular mass index; LVH = Left ventricular hypertrophy; E = early diastolic mitral inflow velocity; e\` = early diastolic mitral annular velocity; LVDD = left ventricular diastolic dysfunction; EF = ejection fraction; LVSD = left ventricular systolic dysfunction; CO, cardiac output; CI cardiac index. d, end diastolic; s, end systolic

Supplementary Table S2. Changes in Echocardiographic Parameters in the Low Qa/CO Group

Parameters	Baseline	1 year later	*p* value
Aortic root	3.10±0.41	3.14±0.40	0.157
LAD, cm	4.11±0.60	4.05±0.59	0.388
LAVI, ml/m^2	45.9±15.7	42.9±14.1	0.035
RVIDd, mm	3.04±0.40	3.07±0.45	0.458
IVSd, mm	1.08±0.18	1.07±0.20	0.510
IVISs, mm	1.41±0.22	1.40±0.23	0.557
LVIDd, mm	4.70±0.66	4.70±0.58	0.962
LVIDs, mm	3.20±0.60	3.22±0.66	0.799
LVPWd, mm	1.07±0.16	1.08±0.26	0.570
LVPWs, mm	1.38±0.19	1.36±0.18	0.369
E, cm/s	93.3±28.4	88.3±27.1	0.054
A	101.1±23.6	98.6±23.2	0.164
E/A	0.96±0.45	0.94±0.51	0.456
RWT, mm	0.46±0.10	0.47±0.14	0.475
RVSP, mmHg	30.4±13.9	29.5±13.8	0.409
LVM, g	186.3±53.6	184.7±55.4	0.545
LVMI, g/m^2	111.3±29.6	111.3±29.8	0.979
E/e` ratio	14.7±5.2	14.0±5.2	0.113
EF, %	58.3±7.7	58.5±7.7	0.715
CO, L/min	5.81±1.53	6.05±2.32	0.287
CI, L/min/m^2	3.50±0.87	3.69±1.46	0.184
Effective CO, L/min	4.90±1.51	5.01±2.22	0.299
Effective CI, L/min/m^2	2.91±0.85	3.10±1.39	0.216
Qa + CO, L/min	6.77±1.62	7.02±2.51	0.288
CO/Qa	0.17±0.06	0.17±0.08	0.798
Qa, ml/min	930.4±342.9	949.9±435.6	0.710

RVID, RV internal dimension; LAD = left atrial dimension; LAVI=left atrial volume index; LVID, left ventricular Internal dimension; IVS, Interventricular septum thickness; PW = posterior wall thickness; RWT, relative wall thickness; RVSP, RV systolic pressure; LVMI = Left ventricular mass index; LVH = Left ventricular hypertrophy; E = early diastolic mitral inflow velocity; e` = early diastolic mitral annular velocity; LVDD = left ventricular diastolic dysfunction; EF = ejection fraction; LVSD = left ventricular systolic dysfunction; CO, cardiac output; CI cardiac index. d, end diastolic; s, end systolic

Supplementary Table S3. Changes in Echocardiographic Parameters According to the Qa/CO Ratio Among Patients with Arteriovenous Fistulas

	Qa/CO ≤ 0.3		*p* value	Qa/CO > 0.3		*p* value
	baseline	1year later		baseline	1year later	
**CO, L/min**	5.72±1.47	5.93±2.16	0.418	5.02±1.31	6.14±2.17	0.005
**CI, L/min/m^2**	3.35±0.74	3.51±1.19	0.292	3.04±0.68	3.70±1.04	0.004
**Effective CO, L/min**	4.79±1.48	4.92±2.04	0.602	3.09±0.97	4.50±1.98	0.002
**Effective CI, L/min/m^2**	2.81±0.77	2.91±1.13	0.492	1.87±0.50	2.71±1.00	0.001
**CO+Qa, L/min**	6.65±1.53	6.93±2.36	0.280	7.15±1.71	8.10±2.41	0.020
**Qa/CO**	0.17±0.07	0.18±0.08	0.560	0.40±0.09	0.31±0.12	<0.001
**Qa,** ml/min	935.2±345.3	986.6±452.0	0.173	2052.2±525.6	1837.4±594.3	0.005

Supplementary Table S4. Changes in Echocardiographic Parameters According to the Qa/CO Ratio Among Patients with Arteriovenous Grafts

	Qa/CO ≤ 0.3		*p* value	Qa/CO > 0.3		*p* value
	baseline	1year later		baseline	1year later	
**CO, L/min**	6.06±1.69	6.40±2.74	0.497	5.16±0.54	5.00±0.79	0.500
**CI, L/min/m^2**	3.88±1.07	4.15±1.95	0.427	3.12±0.29	3.04±0.40	0.500
**Effective CO, L/min**	5.05±1.60	5.60±2.62	0.296	2.94±0.08	3.63±0.15	0.046
**Effective CI, L/min/m^2**	3.24±1.00	3.60±1.83	0.282	1.81±0.05	2.22±0.01	0.037
**CO+Qa, L/min**	7.06±1.83	7.23±2.91	0.747	7.38±1.01	6.33±1.44	0.179
**Qa/CO**	0.17±0.06	0.15±0.09	0.272	0.43±0.05	0.27±0.09	0.114
**Qa,** ml/min	1031.8±328.8	860.7±384.9	0.036	1980.0±433.4	1060.0±504.1	0.004

CO, cardiac output; CI cardiac index
